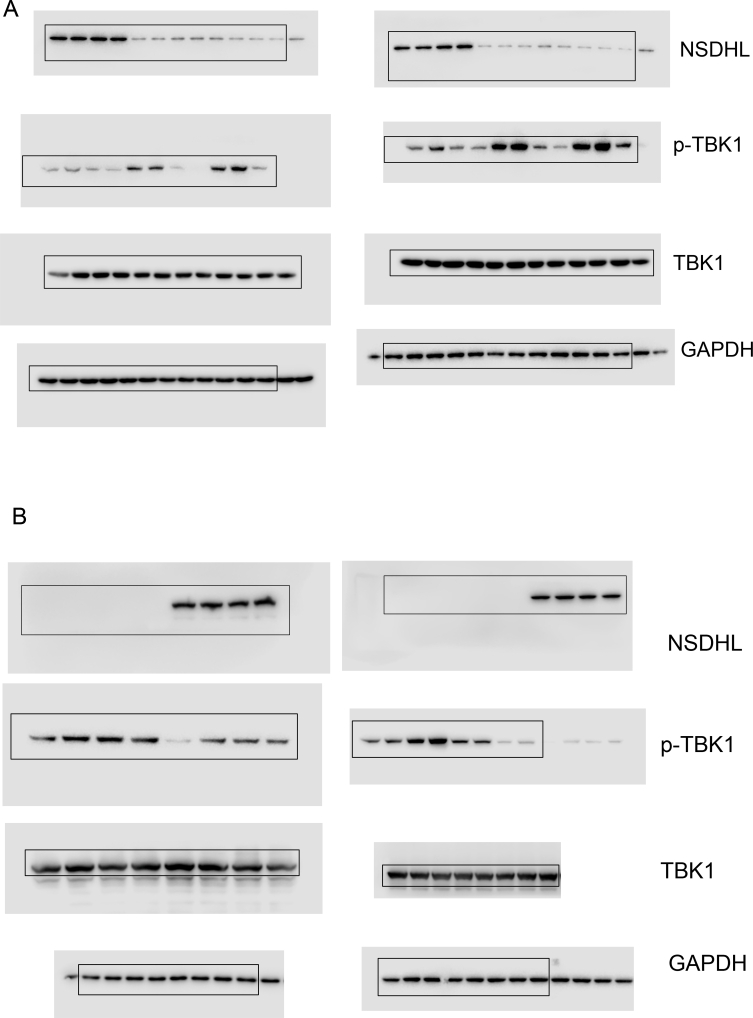# NSDHL promotes the degradation of sting in cholangiocarcinoma

**DOI:** 10.1016/j.heliyon.2024.e37592

**Published:** 2024-09-07

**Authors:** Weihua Yu, Jionghuang Chen, Yifan Tong, Linghua Zhu, Yuezheng Deng, Junju He, Chenxi Zhong, Xiujun Cai

**Affiliations:** a Department of General Surgery, Sir Run Run Shaw Hospital, School of Medicine, Zhejiang University, Hangzhou, 310000, China; b Liver Regeneration and Metabolism Study Group, Sir Run Run Shaw Hospital, School of Medicine, Zhejiang University, Hangzhou, 310000, China; c Shanghai Institute of Thoracic Oncology, Shanghai Chest Hospital, Shanghai Jiao Tong University School of Medicine, Shanghai, 200030, China; d Department of Oncology, Renmin Hospital of Wuhan University, Wuhan University, Wuhan, 430060, China

**Keywords:** NSDHL, Cyclic-GMP-AMP-Synthase, STING, Cholangiocarcinoma

## Abstract

Metabolic enzymes play significant roles in tumor growth via nonmetabolic biological processes. However, more research is needed to understand their roles in immune modulation. This study revealed that 3-hydroxysteroid dehydrogenase (NSDHL) expression was elevated in cholangiocarcinoma. In vitro experiments demonstrated that NSDHL had no effect on the growth or invasion of cholangiocarcinoma cells in an artificial laboratory environment. However, NSDHL overexpression strongly enhanced the promotion of AKT/YAP-driven cholangiocarcinoma. NSDHL bound to STING and facilitated its degradation via ubiquitination. This inhibited the cyclic-GMP-AMP-synthase-STING signaling pathway and reduced the synthesis of IFNβ. A study revealed an inverse relationship between the expression of NSDHL and the infiltration of NK cells, activated CD4^+^ T cells, and neutrophils in individuals who were diagnosed with cholangiocarcinoma. This study elucidates the role of NSDHL, in addition to its established metabolic functions, NSDHL regulates the cyclic-GMP-AMP-synthase signaling pathway. By exploring this interplay, this research enriches our understanding of the functions of NSDHL in terms of cellular dynamics, offering novel insights into the modulation of crucial biological pathways.

## 1 Introduction

Cholangiocarcinoma is a very common malignancy [1]. Scientists are currently using rapamycin and immune checkpoint inhibitors, such as anti-PD-L1 monoclonal antibodies and atni-CTLA4 monoclonal antibodies, to treat cholangiocarcinoma in the clinic [1]. These drugs work by blocking the mTOR signaling pathway and reshaping the immune microenvironment. Specifically, immune checkpoint inhibitors have greatly improved the survival of patients [2]. Nevertheless, immunotherapy has limitations, and increasing the rate of response to immunotherapy is a crucial concern in the field of clinical tumor treatment.

For tumor immunotherapy to be successful, stimulating the Cyclic-GMP-AMP synthase (cGAS) signaling pathway and establishing a proinflammatory immune environment are essential [3]. These pathways are crucial for the response to immunotherapy facilitated by anti-PD-1 antibodies [4]. The cGAS enzyme can identify and recognize unbound DNA molecules within the cytoplasmic region of a cell [5]. cGAS interacts with double-stranded DNA (dsDNA) to catalyze the formation of the second messenger cyclic guanosine monophosphate–adenosine monophosphate (cGAMP). This event initiates the activation of the STING protein, initiating a cascade of signaling pathways that culminate in the production of interferon (IFN) and inflammatory chemokines [5,6].

STING, which is an essential adaptor protein in the innate immune system that identifies DNA present in the cell's cytoplasm, is a crucial player in this process [7]. STING mainly functions as a part of the cGAS-driven IFN pathway [7], where cGAS detects unpaired double-stranded DNA (dsDNA). Upon detection of dsDNA, cGAS produces 2′3′-cGAMP (cGAMP). This cGAMP binds to and activates STING in the endoplasmic reticulum (ER) [8]. Activated STING changes its shape and moves from the ER to the perinuclear compartment. Within the perinuclear region, STING interacts with TANK-binding kinase 1 (TBK1). TBK1 subsequently phosphorylates interferon regulatory factor 3 (IRF3). This phosphorylation prompts IRF3 to translocate into the nucleus. In the nucleus, IRF3 binds to the promoters of type I interferons (IFNs) and promotes the expression of interferons, which activate the immune response and protect against viruses and other pathogens. Inflammatory chemokines recruit immune cells to the location of infection, helping to control infection. The cGAS-STING pathway is a critical part of innate immunity, and without this pathway, the immune response can be impaired. The cGAS-STING pathway has been studied for many years, and these studies have led to a better understanding of this pathway and the development of new treatment methods [9].

A major characteristic, namely, metabolic reprogramming, distinguishes tumor cells from normal cells [10]. Cancer, such as pancreatic cancer and breast cancer, significantly stimulates cholesterol synthesis [11]. Many types of cancers increase the activity of HMGCR, which is the enzyme that controls the rate of cholesterol production [11]. The NSDHL gene encodes the enzyme 3β-hydroxysteroid dehydrogenase, which plays a crucial role in the cholesterol biosynthesis process [11]. Recent studies have shown that NSDHL plays a significant role in promoting the spread of triple-negative breast cancer, making it a possible driver of metastasis [12]. NSDHL expression is substantially increased in breast cancer tissues and serves as a reliable indicator of an unfavorable prognosis [12]. Suppression of NSDHL significantly hinders cell proliferation and migration [12]. By preventing the breakdown of TGFβR2 in endosomes, NSDHL activates the TGFβ signaling pathway [12]. Nevertheless, the specific function of NSDHL, as well as its underlying mechanisms, in tumors are not fully understood.

The objective of this work was to examine the NSDHL expression pattern in cholangiocarcinoma and elucidate its role and associated mechanisms in the progression of cholangiocarcinoma.

## 2 Materials and methods

### 2.1 Cell culture, clinical samples and ethical permission

Cholangiocarcinoma and normal biliary epithelial (HIBEpiC) cell lines were obtained from the Cell Bank of the Chinese Academy of Sciences and subsequently nurtured in DMEM. This medium was supplemented with 10 % fetal bovine serum, 100 U/mL penicillin and 100 μg/mL streptomycin to ensure optimal growth and sterility. The incubation conditions were set to 5 % CO_2_ and 37 °C. Lipofectamine 8000 was used for transfection. For analysis of NSDHL expression, we obtained clear consent from our study participants for the clinical samples. Additionally, the use of these samples in our research received approval from the Ethics Committee of Zhejiang University.

### 2.2 Immunohistochemistry

The tissue sections were dewaxed, rehydrated and heated in EDTA solution for 30 min for antigen retrieval. Once the samples had cooled to room temperature, a natural peroxidase inhibitor was used via incubation for 15 min. Next, we washed the sample 1–2 times with phosphate-buffered saline (PBS). The tissue samples were treated with anti-NSDHL or anti-ki67 antibodies at 4 °C overnight. The next day, we washed the tissue sections three times with PBS before they were incubated with secondary antibodies at room temperature for 2 h. We used 3,3′-diaminobenzidine (DAB) for visualization via immunohistochemistry. After hematoxylin was used to counterstain all the tissue samples, the staining strength and protein expression were automatically assessed via Vectra2.0 system.

### 2.3 Western blotting

The cells were washed twice with PBS and then lysed with RIPA buffer containing phosphatase and protease inhibitors. Thereafter, the sample was centrifuged to separate the supernatant, and after the supernatant was collected, the protein concentration was quantified via a BCA Kit. SDS‒PAGE was used to separate the proteins. The isolated proteins were transferred to PVDF membranes, which were incubated with specific antibodies overnight at 4 °C. After incubation, the membranes were further incubated with HRP-conjugated secondary antibodies for 1–2 h. The present study included the antibodies targeting the following proteins: NSDHL (Proteintech, 15111-1-AP, 1:1000); tubulin (Santa Cruz Biotechnology, sc-5286, 1:4000); Flag (Proteintech, 66,008-4-lG, 1:1000); GAPDH (Proteintech, 10494-1-AP, 1:1000); GST-tag (Abcam, ab307273, 1:1000); Sting (Proteintech, 19851-1-AP, 1:1000); HA (Proteintech, 66006-2-Ig, 1:1000); CBL (Proteintech, 25818-1-AP, 1:1000); p-TBK1 (Abcam, ab186469, 1:1000); and TBK1 (Proteintech, 28397-1-AP, 1:1000).

### 2.4 Overexpression or knockdown of NSDHL

shRNA was designed via an online tool available on Sigma's website. The shRNAs specifically targeted NSDHL and CBS. We selected the pLKO.1 plasmid for cloning. The lentivirus was packaged in 293T cells with the vectors PMD2.G. and psPAX2. The virus that had been collected was subsequently condensed via PEG800 and subjected to centrifugation (1600×*g*) for 1 h (4 °C). The virus was dissolved in DMEM. Then, the cells were seeded in a six-well plate. On the following day, a volume of 400 μL of lentivirus was introduced and allowed to incubate with the cells overnight at 37 °C. Following a two-day interval, the culture was supplemented with puromycin (1 μg/mL) and incubated for an additional four days. The purpose of this process was to select stable cell lines. Finally, the expression levels of NSDHL and CBL were determined via western blotting.

### 2.5 Crystal violet assay

A crystal violet assay was used to monitor cell growth. To do this, 10^3^ cells were seeded in 48-well plates and cultured for seven consecutive days in medium containing 1 % FBS. We changed the medium every other day to ensure maximum cell proliferation. Furthermore, we replaced the media with 20 % methanol and 0.5 % crystal violet solution, and the cells were incubated with crystal violet solution for 5 min. Next, they were washed with phosphate-buffered saline and then photographed.

### 2.6 Transwell assay

65 μl of the Matrigel-RPMI-1640 media mixture (v:v, 100:3) was applied to the bottom membrane of the top chamber. We then allowed the solidification of the mixture at 37 °C for 30 min. A culture mixture containing 0.1 % FBS was used to prepare the cell suspension, which was adjusted to a concentration of 5˟ 10^5^ cells/mL. 200 μl of cell suspension was added into the upper chambers, and 500 μl of 30 % fetal bovine serum was added to the bottom chambers. We incubated the plate for 60 h at 37 °C in the presence of 5 % CO_2_. Thereafter, the chamber membranes were fixed with 4 % paraformaldehyde and stained with 0.3 % crystal violet solution after being washed three times in PBS. After five randomly chosen fields of view were observed via a Leica DMI4000B microscope at a magnification of 20 × , images of the cells were captured and quantified via the online tool ImageJ.

### 2.7 Hydrodynamic model

Intrahepatic cholangiocarcinoma was established in C57BL/6J mice via hydrodynamic injection. The 0.9 % NaCl solution was filtered and used to dilute the pT3-EF1a-HA-myr-AKT, pT3-EF1a-Yap^S127A^, and pT3-EF1a-NSDHL plasmids. The mice were administered 20 μg of pT3-EF1a-HA-myr-AKT, 30 μg of pT3-EF1a-Yap^S127A^, or either the control or NSDHL expression plasmid for 5–7 s via the tail vein.

### 2.8 Immunoprecipitation

IP lysis buffer containing protease and phosphatase inhibitors was used to harvest the cells. The lysate was centrifuged, and the supernatant was treated with 1 μg of antibody and incubated at 4 °C overnight. The next day, 40 μL of protein A/G beads (B23202, Bimake.com) were added to the supernatant and incubated at 4 °C for 2 h. Before the beads were loaded with loading buffer for western blot analysis, they were washed three times with wash buffer.

### 2.9 GST pull-down

10 μg of the GST-STING fusion protein was added to the supernatants of the cell lysates. The incubation was carried out overnight at 4 °C, allowing sufficient time for the GST-tagged protein to effectively bind and capture interacting proteins from the lysate. After incubation, the glutathione Sepharose 4B beads were added for another incubation of 4 h. These beads were subjected to three rigorous washes with buffer containing 50 mM Tris-HCl (pH 8.0), 150 mM NaCl, and 1 % NP-40 to eliminate nonspecific proteins and contaminants. After been washing, the beads were prepared for analysis by adding 1 × loading buffer and heating at 100 °C for 5 min to denature the proteins, after which they were released into the supernatant. This protein-rich supernatant was then collected for western blotting, which enabled us to identify the specific protein‒protein interactions involved in our study.

### 2.10 qPCR

RNA was extracted via TRIzol. 1 μg of RNA was reverse transcribed into complementary DNA via the PrimeScriptTM RT Kit (Takara). This qPCR experiment was performed with SYBR Green Reagent and a CFX96 real-time fluorescence quantitative PCR detection instrument (Bio-Rad, Richmond, CA, USA).

### 2.11 HT-DNA treatment

The cells were first seeded in six-well plates, and 24 h later, the HT-DNA was transfected into the cholangiocarcinoma cells via Lipofectamine 3000. Western blotting was used to identify TBK1 phosphorylation after the cells were harvested at different time points.

### 2.12 E3 substrate prediction and correlation analysis

The E3 of the substrate was predicted via UbiBrowser, which is available at the online portal ncpsb (http://ubibrowser.ncpsb.org). By combining the substrate gene with the ten most confidently predicted E3, an E3-substrate interaction network was built. To analyze and visualize the networks, Cytoscape v3.4.0 (https://cytoscape.org/) was used. The associations were examined via Pearson correlation analysis. A p value less than 0.05 was considered significant.

### 2.13 Statistical methods

The experimental results are reported as the mean values plus or minus the standard deviation. Data analysis was performed via t tests. Next, we used a log-rank test to analyze the survival curves generated by the Kaplan‒Meier technique. Statisticians used GraphPad Prism 8 and 17.0 versions of SPSS.

## 3 Results

### 3.1 Upregulation of NSDHL in cholangiocarcinoma

In this study, we first compared NSDHL expression in cholangiocarcinoma cells and normal cholangiocytes (HIBEpiCs). Western blotting analysis of various cholangiocarcinoma cell lines (RBE, HCCC9810, HUCCT1, TFK1, and SNU869) revealed increased NSDHL levels (Fig. 1A). Additionally, NSDHL expression was assessed in six cholangiocarcinoma tissues and their adjacent noncancerous counterparts, and the results revealed increased NSDHL expression in the cancerous tissues (Fig. 1B). Immunohistochemical analysis of 46 cholangiocarcinoma tissues confirmed elevated NSDHL expression (Fig. 1C). In addition, high NSDHL expression was correlated with reduced survival in cholangiocarcinoma patients in the GEPIA database (Fig. 1D), emphasizing its potential role in the progression of cholangiocarcinoma.

**Fig. 1:**
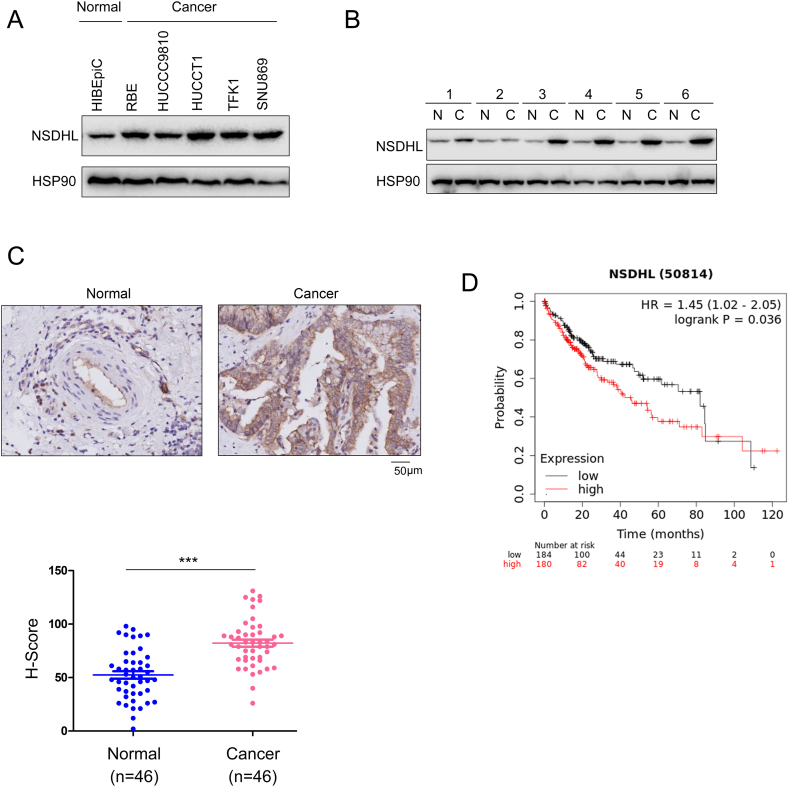
**NSDHL is upregulated in cholangiocarcinoma.** (A) NSDHL expression in normal cholangiocytes and cholangiocarcinoma cells detected by Western blotting (The original image is provided in the Supplementary Fig. S1A). (B) Protein levels of NSDHL in adjacent nontumor tissue (N) and cholangiocarcinoma tissue (C) detected by Western blotting (The original image is provided in the Supplementary Fig. S1B). (C) Protein levels of NSDHL in adjacent nontumor tissue (normal) and cholangiocarcinoma tissue detected by IHC, with statistical analysis. (D) Analysis of the relationship between NSDHL expression and survival of cholangiocarcinoma patients using the Human Protein Atlas (HPA) database. ***, *P* < 0.001.

### 3.2 NSDHL promotes the occurrence of cholangiocarcinoma

To determine whether NSDHL promotes the occurrence of cholangiocarcinoma, we altered the expression of NSDHL in various cholangiocarcinoma cell lines. NSDHL expression was overexpressed in the HUCCC9810 and TFK1 cell lines but knocked down in the RBE and HUCCT1 cell lines (Fig. 2A and B). These changes did not affect cell growth in liquid culture medium (Fig. 2C). Additionally, increased expression of NSDHL does not affect the invasive capacity of cholangiocarcinoma cells (Fig. 2D). However, we found that the expression of NSDHL in mouse cholangiocytes promoted the AKT- and YAP^S127A^-induced development of cholangiocarcinoma (Fig. 2E), and elevated the number of Ki67-positive cells. These results suggest that increased expression of NSDHL promotes the progression of cholangiocarcinoma possibly by regulating the tumor microenvironment.

**Fig. 2:**
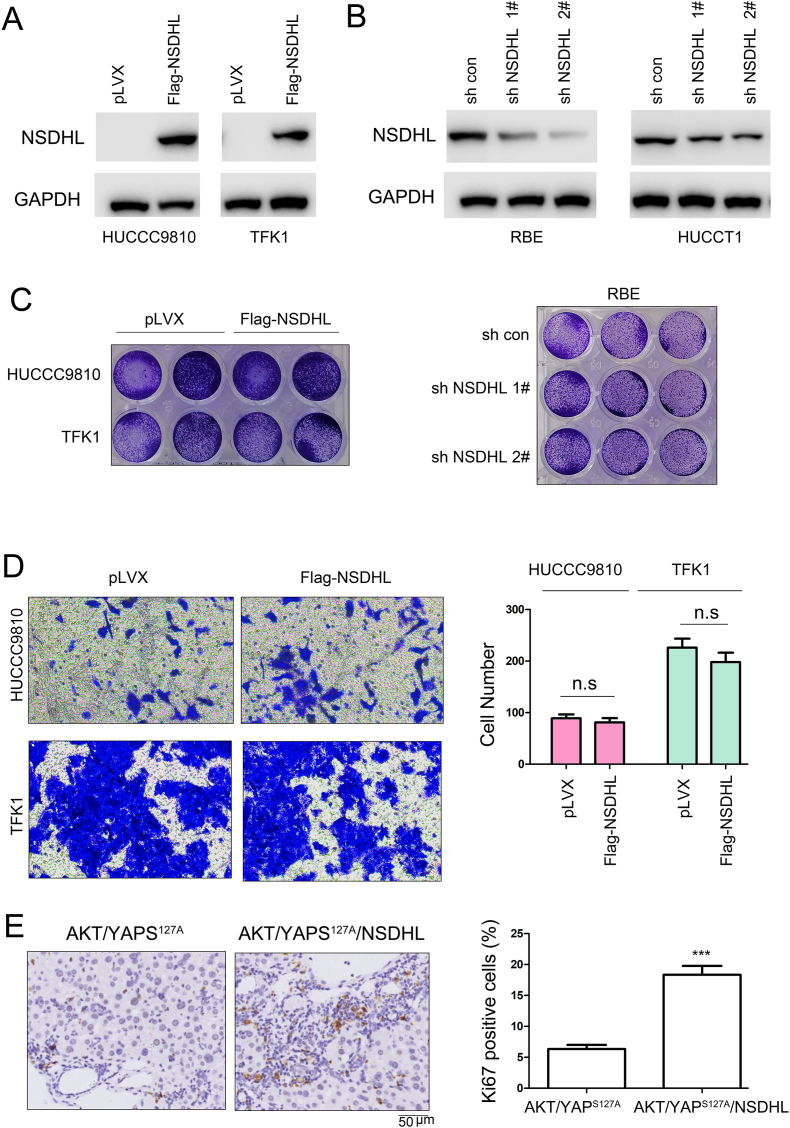
**The expression of NSDHL has no significant effect on the growth and invasion of cholangiocarcinoma but promotes the occurrence of cholangiocarcinoma in mice.** (A) Overexpression of exogenous NSDHL (Flag-NSDHL) in cholangiocarcinoma cells detected by Western blotting (The original image is provided in the Supplementary Fig. S2A). (B) Knockdown of endogenous NSDHL expression in cholangiocarcinoma cells detected by Western blotting (The original image is provided in the Supplementary Fig. S2B). (C) Effects of NSDHL overexpression and NSDHL knockdown on the in vitro growth of cholangiocarcinoma cells detected by crystal violet assay. (D) Effects of NSDHL overexpression on the invasion of cholangiocarcinoma cells detected by Transwell assay and statistically analyzed. (E) Effects of NSDHL overexpression on the occurrence of cholangiocarcinoma in hydrodynamic mouse model, with the proportion of Ki67-positive cells statistically analyzed. Scale bar: 50 μm. ***, *P* < 0.001.

### 3.3 NSDHL interacts with STING

An important part of the tumor immune microenvironment is shaped by the cGAS–STING signaling pathway. We then conducted a comprehensive investigation of the interaction between NSDHL and essential components of the cGAS-STING signaling pathway to evaluate the involvement of NSDHL in this pathway. Flag-NSDHL and HA-STING interact with each other, as shown in Fig. 3A. The results of the GST pull-down experiments shown in Fig. 3B revealed the successful binding of the GST-STING fusion protein with endogenously expressed NSDHL (Fig. 3B). Moreover, a complex was formed between endogenously expressed NSDHL and STING (Fig. 3C). Notably, the activation of cGAS-STING signaling via HT-DNA disrupted the interaction between NSDHL and STING (Fig. 3D). Notably, the interaction between NSDHL and STING was mediated by the C-terminus of the STING protein (Fig. 3E), as shown in Fig. 3E. These observations clearly indicate that NSDHL and STING interact.

**Fig. 3:**
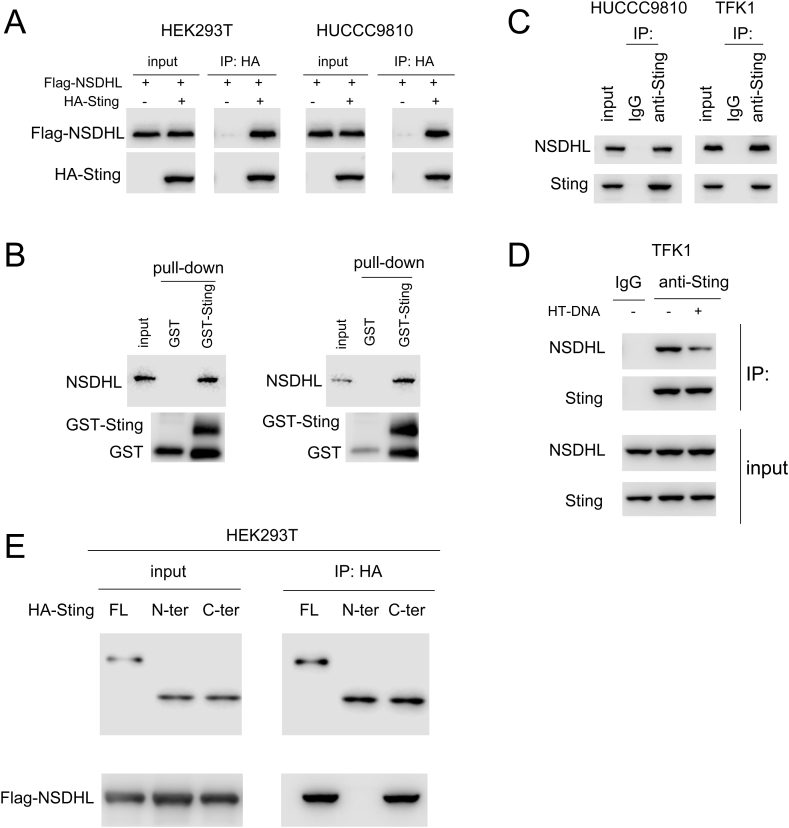
**Interaction between STING and NSDHL.** (A) An immunoprecipitation assay was performed to detect the interaction between exogenously expressed STING (HA-STING) and NSDHL (Flag-NSDHL) (The original image is provided in the Supplementary Fig. S3A). (B) GST-pulldown assay was conducted to examine the interaction between fusion protein GST-STING and endogenously expressed NSDHL in cholangiocarcinoma cells (The original image is provided in the Supplementary Fig. S3B). (C) Immunoprecipitation assay was employed to detect the interaction between endogenously expressed STING and NSDHL in cholangiocarcinoma cells (The original image is provided in the Supplementary Fig. S3C). (D) Immunoprecipitation was performed to determine the effect of HT-DNA stimulation on the interaction between STING and NSDHL. Cholangiocarcinoma cells were transfected with HT-DNA (4 μg), and after 4 h, cell lysates were collected and subjected to immunoprecipitation using an anti-STING antibody (The original image is provided in the Supplementary Fig. S3D). (E) Immunoprecipitation was performed to identify the region responsible for the interaction between STING protein and NSDHL (The original image is provided in the Supplementary Fig. S3E).

### 3.4 NSDHL promotes the CBL-mediated ubiquitination and degradation of STING

We then investigated the regulatory effect of NSDHL on STING in detail. Fig. 4A shows that NSDHL overexpression caused a decrease in STING protein levels, and this decrease was dose dependent. It was hypothesized that NSDHL facilitates the degradation of STING via the ubiquitination pathway. Our investigation of the UbiBrowser database indicated that CBL may act as an E3 ubiquitin ligase for STING, as shown in Fig. 4B. An immunoprecipitation assay (Fig. 4C) revealed the interaction between exogenously expressed STING and CBL. In the GST pull-down study, the GST-STING fusion protein effectively captured the endogenously expressed CBL protein in cholangiocarcinoma cells (Fig. 4D). Additionally, we demonstrated complex binding between naturally expressed STING and CBL, as shown in Fig. 4E. Next, we investigated the effect of NSDHL expression on the interaction between STING and CBL. Fig. 4F shows that NSDHL facilitated the interaction between STING and CBL. Moreover, as shown in Fig. 4G, STING underwent CBL-mediated ubiquitination.

**Fig. 4:**
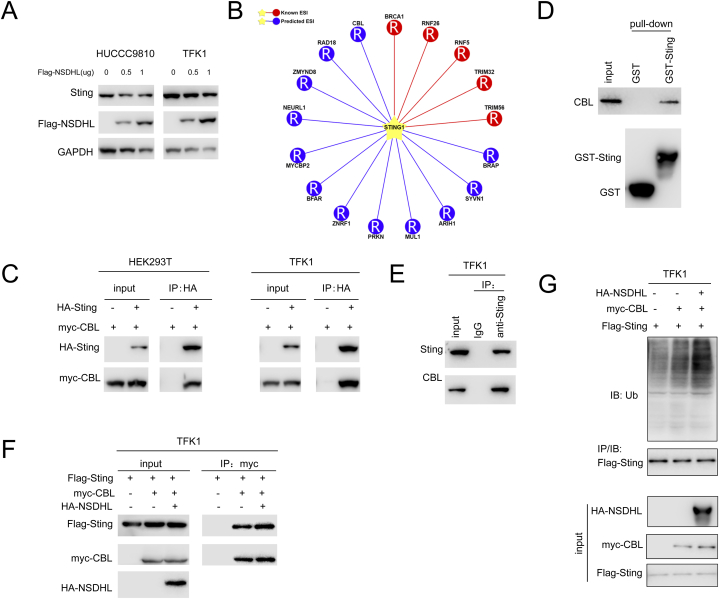
**NSDHL promotes the degradation of STING through the ubiquitination pathway.** (A) The effect of exogenously overexpressed NSDHL (Flag-NSDHL) on the protein level of endogenously expressed STING was detected using Western blotting (The original image is provided in the Supplementary Fig. S4A). (B) The E3 ligase for STING was predicted using the UbiBrowser database, and CBL was identified as a potential E3 ligase for STING. (C) The interaction between exogenously expressed STING (HA-STING) and CBL (myc-CBL) was detected using immunoprecipitation experiments (The original image is provided in the Supplementary Fig. S4C). (D) The interaction between the fusion protein GST- STING and endogenously expressed CBL in cholangiocarcinoma cells was detected using GST-pulldown experiments (The original image is provided in the Supplementary Fig. S4D). (E) Immunoprecipitation experiments were performed using anti- STING antibodies to detect the interaction between endogenously expressed STING and CBL in cholangiocarcinoma cells (The original image is provided in the Supplementary Fig. S4E). (F) The effect of overexpressed NSDHL (HA-NSDHL) on the interaction between exogenously expressed STING (Flag-STING) and CBL (myc-TRIM41) was detected using immunoprecipitation experiments (The original image is provided in the Supplementary Fig. S4F). (G) The effect of NSDHL expression on the ubiquitination level of STING was detected using Ubiquitination assays (The original image is provided in the Supplementary Fig. S4G).

### 3.5 Inhibition of the cGAS-STING signaling pathway by NSDHL

**Fig. 5:**
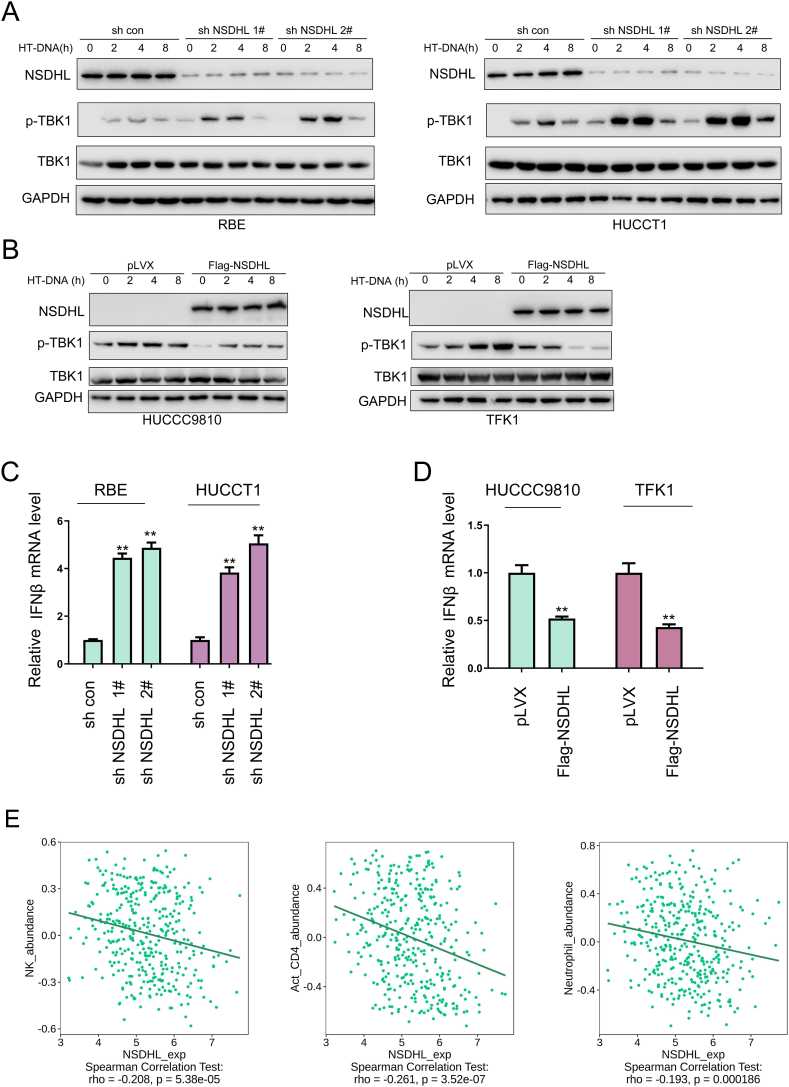
**NSDHL inhibits the cGAS-STING signaling pathway.** (A) The effect of NSDHL knockdown on the phosphorylation of TBK1 at S172 was detected using Western blotting (The original image is provided in the Supplementary Fig. S5A). (B) The impact of NSDHL overexpression on the phosphorylation of TBK1 at S172 was detected in cholangiocarcinoma cells stimulated with HT-DNA (The original image is provided in the Supplementary Fig. S5B). (C) The effect of NSDHL knockdown on the expression of IFNβ was detected using qPCR. (D) The effect of NSDHL overexpression on the expression of IFNβ was detected using qPCR. (E) The correlations between NSDHL expression and the infiltration of the immune cells were analyzed using the public database. ***, *P* < 0.01.

Next, we investigated the influence of NSDHL expression on the activation of the cyclic GMP-AMP synthase pathway. The knockdown of NSDHL promoted HT-DNA-induced TBK1 phosphorylation, as shown in Fig. 5A. In contrast, overexpression of NSDHL inhibited TBK1 phosphorylation, as shown in Fig. 5B. IFNβ was identified as a gene that is activated as a result of the cGAS–STING signaling pathway. Additionally, knockdown of NSDHL increased the mRNA level of IFNβ (Fig. 5C), whereas overexpression of NSDHL resulted in a decrease in the IFNβ mRNA level (Fig. 5D). Moreover, we observed a decrease in the infiltration of NK cells, activated CD4^+^ T cells, and neutrophils when the expression of NSDHL was upregulated (Fig. 5E). These results suggested that NSDHL effectively inhibited the cGAS‒STING signaling pathway.

## 4 Discussion

The significant trait of metabolic reprogramming distinguishes tumor cells from normal cells [13]. Tumor tissues exhibit significant upregulation of metabolic enzymes, particularly those associated with glycolysis and serine production [14,15]. Emerging new information suggests that some metabolic enzymes that help control metabolism, such as PKM2, PGK1, HK2, and PCK1, also perform nonmetabolic-related functions [14]. The nonmetabolic roles of these enzymes are crucial in tumor progression [16]. Exploiting the nonmetabolic actions of these enzymes holds great promise for tumor therapy. This study revealed an increase in NSDHL in cholangiocarcinoma cells, but it did not affect their malignant characteristics in a laboratory setting. However, overexpressing NSDHL in the livers of C57BL/6 mice with fully functional immune systems greatly accelerated the growth of cholangiocarcinoma. This discovery suggests a strong link between NSDHL and immunological modulation. Through molecular biology research, we discovered that NSDHL engages in interactions with STING, which is a pivotal molecule in the innate immune response, and NSDHL inhibits the production of IFNβ, thereby impeding immune cell infiltration.

The identification of the regulatory role of NSDHL in the cGAS-STING pathway is a significant finding from this study. Tumor immunotherapy benefits from the inflammatory effects of the cGAS-Sting pathway [17]. There is a dearth of studies on metabolic enzymes that directly impact immune regulation [18]. Our knowledge of the biological importance of NSDHL and the idea of a “metabolic state in immune regulation” were both increased in this work. Furthermore, the relationship between the control of STING stability by NSDHL and its substrate or product remains unclear and requires further investigation.

This study represents a significant advancement in the understanding of STING protein regulation, elucidating key molecular mechanisms that govern its stability and activity. Posttranslational modifications such as phosphorylation and acetylation reportedly modulate the interactions of STING with DNA and its propensity for dimerization [19]. The discovery that RNF144A facilitates STING ubiquitination and subsequent degradation, with USP13 acting in opposition to reversing this process, highlights a finely regulated regulatory mechanism [20,21]. Importantly, the involvement of CBL in STING degradation further delineates the pathways through which the stability of STING is maintained. Moreover, this study revealed that the interaction between STING and NSDHL is weakened after activation of the cGAS-STING pathway by HT-DNA. This interaction signifies the role of NSDHL as a modulator of the cGAS-STING signaling pathway, which impacts cellular responses to DNA damage and immune activation.

Finally, these findings not only increase our understanding of STING regulation but also offer promising avenues for therapeutic intervention, particularly in diseases where STING dysregulation plays a critical role, such as cholangiocarcinoma. The identification of NSDHL as a key player in this regulatory network suggests new strategies for drug development aimed at modulating STING activity and improving treatment outcomes for related disorders. This study thus contributes valuable insights to the scientific community, advancing knowledge of cellular signaling and paving the way for future research into targeted therapies.

## Data availability statement

Data will be provided upon request.

## CRediT authorship contribution statement

**Weihua Yu:** Writing – review & editing, Writing – original draft, Visualization, Validation, Conceptualization. **Jionghuang Chen:** Writing – review & editing, Writing – original draft, Validation, Project administration, Funding acquisition, Formal analysis. **Yifan Tong:** Writing – original draft, Visualization, Validation. **Linghua Zhu:** Writing – review & editing, Validation. **Yuezheng Deng:** Writing – review & editing, Validation, Supervision, Formal analysis. **Junju He:** Writing – review & editing, Supervision, Resources. **Chenxi Zhong:** Writing – review & editing, Methodology. **Xiujun Cai:** Writing – review & editing, Conceptualization.

## Declaration of competing interest

The authors declare the following financial interests/personal relationships which may be considered as potential competing interests:Jionghuang Chen reports financial support was provided by 10.13039/501100004731Zhejiang Provincial Natural Science Foundation of China. If there are other authors, they declare that they have no known competing financial interests or personal relationships that could have appeared to influence the work reported in this paper.

## Figures and Tables

**figs1:**
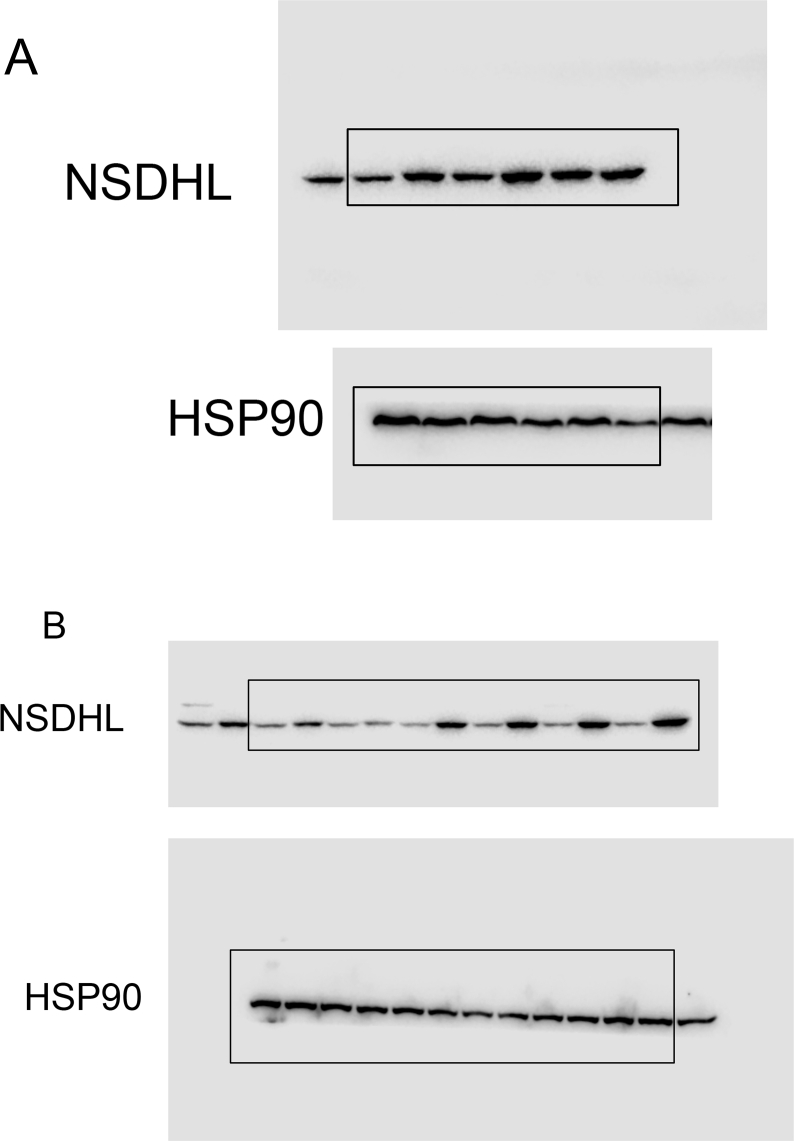


**figs2:**
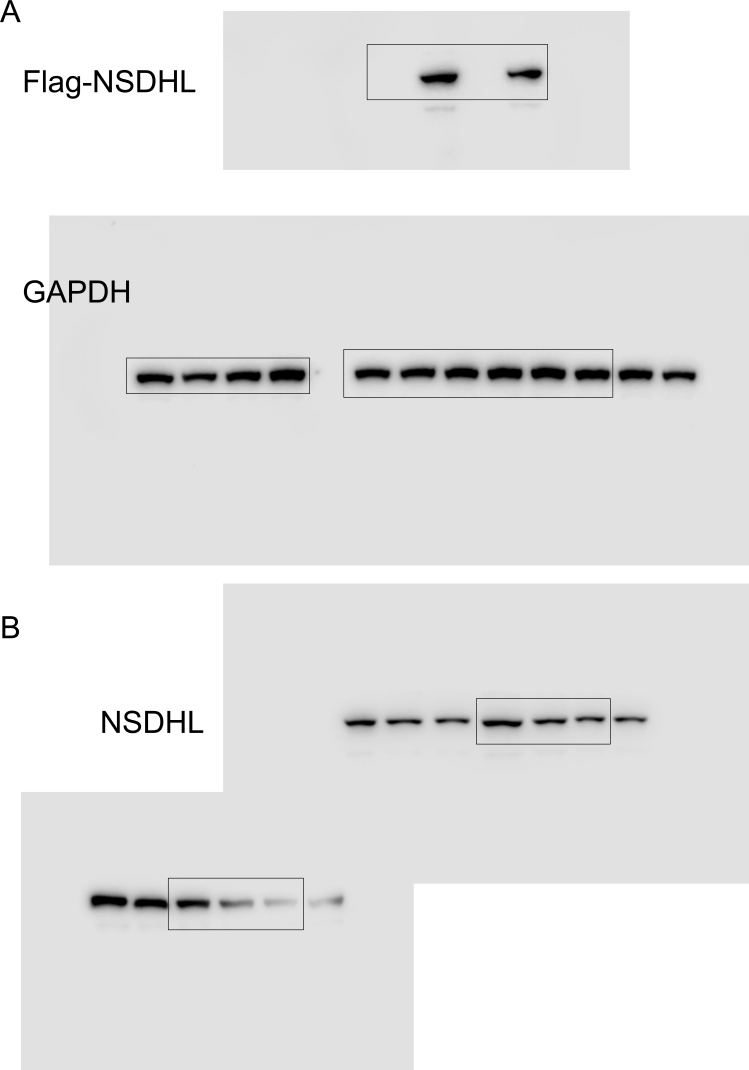


**figs3:**
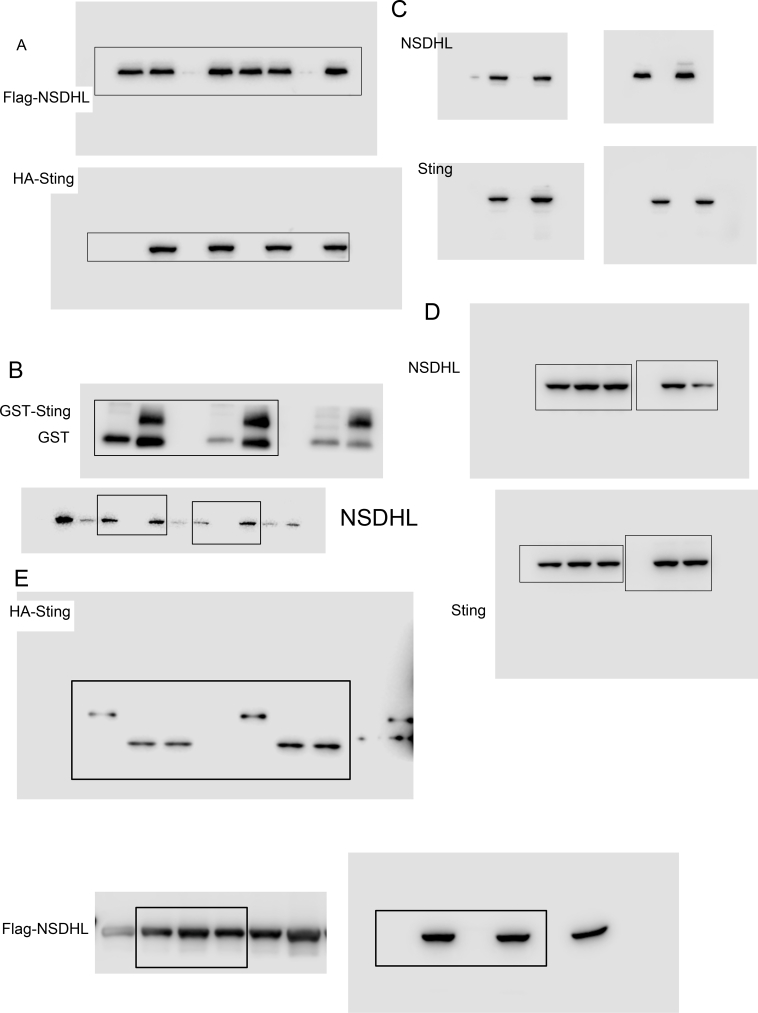


**figs4:**
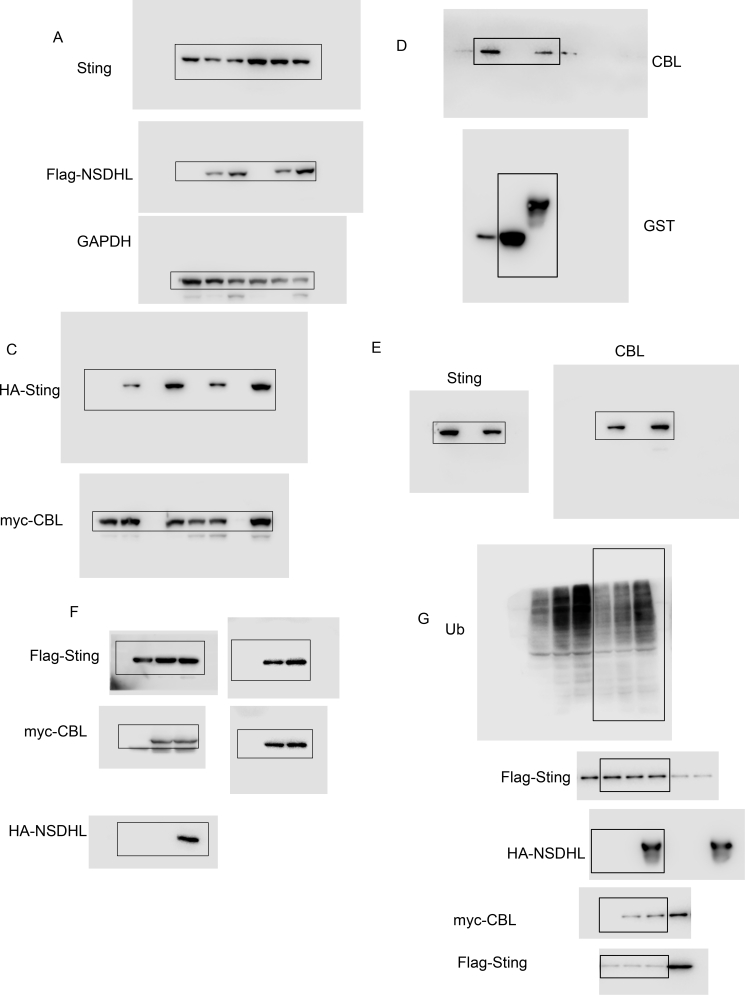


**figs5:**